# Diffusion weighted MRI is a promising imaging biomarker in brain metastases

**DOI:** 10.1007/s11060-014-1642-8

**Published:** 2014-10-30

**Authors:** R. Zakaria, M. D. Jenkinson

**Affiliations:** 1 The Walton Centre NHS Foundation Trust, Liverpool, UK; 2 Institute of Integrative Biology, University of Liverpool, Liverpool, UK

To the Editor,

We read with great interest the article “Application of diffusion-weighted magnetic resonance imaging to predict the intracranial metastatic tumor response to gamma knife radiosurgery” by Lee, Wintermark, Xu, Yen, Schlesinger and Sheehan (J Neurooncol. 2014 Jun; 118(2):351–61). Our group has recently undertaken investigation of DWI/ADC features in resected brain metastases in order to relate these to tissue characteristics and clinical outcomes. The authors present a series of mixed tumor types and add to the increasing evidence that DWI/ADC is a promising marker in these and other brain tumours, due to its ease of acquisition (both in terms of time on the scanner and cost) and robust, logical relation to tissue features such as cellularity.

Brain metastasis studies of all varieties are made difficult by the variable behaviour of tumors depending on their primary cancer of origin. The authors suggest “further studies are required to assess whether DWI/ADC changes are influenced by type of primary cancer.” In fact we have determined as previous researchers have noted (1) that ADC values of brain metastases do indeed differ depending on the primary cancer although this may not be sufficient to distinguish the primary pre-operatively as it is such a crude measure. Rather, values may be lower for more cellular, undifferentiated tumors such as small cell lung cancer compared to better differentiated types such as breast and lung adenocarcinoma (2). We found, however, that this did not limit the applicability of ADC as a predictor of outcomes after surgery and the predictive relationship was maintained even when analysing tumors from a single primary cancer type, in this case lung adenocarcinoma.

Second, the authors note that the “the placement of the ROI ··· relies upon the skill as well as the subjectivity of the authors”. We have also found this practical issue to be a problem and it may well deter clinical radiologists from using such techniques routinely. In support of wider clinical use, we assessed a number of ADC/DWI metrics for a small series of brain metastases and confirmed good inter- and intra-observer agreement when various measures were taken by a senior neuroradiologist and a neuroscience researcher as well as good agreement using different clinically available workstations for post-processing(3). Similar studies have been performed in other organs and other tumors with differing results and more work is clearly needed.

We look forward to further larger studies validating DWI/ADC imaging markers as predictors of outcomes for brain metastases, be it the response to SRS or the survival after surgery. The value of a simple measure, which can be obtained from a widely available sequence, should not be underestimated.

Many thanks for this important study,

Yours sincerely,

Dr R Zakaria MA, Clinical Research Fellow in Neurosurgery, The Walton Centre NHS Foundation Trust & Institute of Integrative Biology, University of Liverpool, UK.

Mr MD Jenkinson PhD, The Walton Centre NHS Foundation Trust & University of Liverpool, UK.
